# Intratumoral administration of mRNA encoding a fusokine consisting of IFN-β and the ectodomain of the TGF-β receptor II potentiates antitumor immunity

**DOI:** 10.18632/oncotarget.2463

**Published:** 2014-11-03

**Authors:** Kevin Van der Jeught, Patrick Tjok Joe, Lukasz Bialkowski, Carlo Heirman, Lidia Daszkiewicz, Therese Liechtenstein, David Escors, Kris Thielemans, Karine Breckpot

**Affiliations:** ^1^ Laboratory of Molecular and Cellular Therapy, Department of Biomedical Sciences, Vrije Universiteit Brussel, Brussels, Belgium; ^2^ Rayne Institute, University College London, London, UK; ^3^ Biomedical Research Centre NavarraBiomed-Fundacion Miguel Servet, National Health Service of Navarre, Pamplona, Navarre, Spain

**Keywords:** mRNA, IFN-β, TGF-β, cancer therapy, T cell

## Abstract

It is generally accepted that the success of immunotherapy depends on the presence of tumor-specific CD8^+^ cytotoxic T cells and the modulation of the tumor environment. In this study, we validated mRNA encoding soluble factors as a tool to modulate the tumor microenvironment to potentiate infiltration of tumor-specific T cells. Intratumoral delivery of mRNA encoding a fusion protein consisting of interferon-β and the ectodomain of the transforming growth factor-β receptor II, referred to as Fβ^2^, showed therapeutic potential. The treatment efficacy was dependent on CD8^+^ T cells and could be improved through blockade of PD-1/PD-L1 interactions. *In vitro* studies revealed that administration of Fβ^2^ to tumor cells resulted in a reduced proliferation and increased expression of MHC I but also PD-L1. Importantly, Fβ^2^ enhanced the antigen presenting capacity of dendritic cells, whilst reducing the suppressive activity of myeloid-derived suppressor cells. In conclusion, these data suggest that intratumoral delivery of mRNA encoding soluble proteins, such as Fβ^2^, can modulate the tumor microenvironment, leading to effective antitumor T cell responses, which can be further potentiated through combination therapy.

## INTRODUCTION

Effective antitumor immune responses rely on the presence and functionality of T cells that are able to recognize and destroy tumor cells. Tumor-specific cytotoxic T lymphocytes (CTLs) are often present in the periphery and within the tumor nest of cancer patients. However, tumor-infiltrating CTLs frequently lack functional antitumor reactivity. The latter is explained by the hostile tumor environment, which is enriched with immunosuppressive cell types, such as immature myeloid cells as well as immunosuppressive factors, including transforming growth factor-β (TGF-β). These inhibitory mechanisms actively quench the antitumor immune response [1, 2].

Over the years, the injection of various antitumor agents such as cells, proteins, nucleic acids or viral vectors, into the tumor has been thoroughly evaluated in preclinical and clinical immunotherapy studies [3]. These agents differ in their mechanism of action: some lead to (re-)activation of effector immune cells, while others counteract the immunosuppressive environment. Strategies developed to induce immune activation include the direct injection of dendritic cells (DCs), and use of DC-recruiting and/or DC-potentiating factors. Our group recently demonstrated that DCs within the tumor have the capacity to engulf mRNA [4–6]. More importantly, we showed that intratumoral delivery of mRNA encoding DC-modulating stimuli, collectively referred to as TriMix, had a therapeutic effect in tumor bearing mice [4]. In the context of the tumor environment, several therapies targeting immunosuppressive mediators such as myeloid-derived suppressor cells (MDSCs), regulatory T (Treg) cells, and secreted factors like TGF-β have been used. Moreover, fusokines such as the FIST protein, consisting of interleukin-2 (IL-2) and the ectodomain of the TGF-β receptor II, have been developed to combine immune activation and tumor modulation [7, 8]. In addition, it was shown that besides activation of CTLs, modulation of the tumor environment is a prerequisite to support the effector phase of the antitumor immune response.

Given above described factors, we evaluated whether we can exploit tumor-residing DCs as ‘factories’ for the production of a mRNA-encoded fusokine. This novel fusokine consists of interferon-β (IFN-β) and the ectodomain of the TGF-β receptor II, and is referred to as Fβ^2^. The rationale for fusing IFN-β with a TGF-β antagonist is based on the observation that blockade of TGF-β within the tumor is most effective when combined with an immune activator [9]. In addition, type I IFNs, such as IFN-β are potent activators of adaptive immune responses within the tumor environment [10]. We demonstrate that the fusokine Fβ^2^ modulates various cell populations, including tumor cells, DCs, MDSCs and CD8^+^ T cells, leading to the favorable therapeutic outcome after its intratumoral delivery in the form of mRNA.

## RESULTS

### Fβ^2^ mRNA is translated into a functional protein

**Figure 1:**
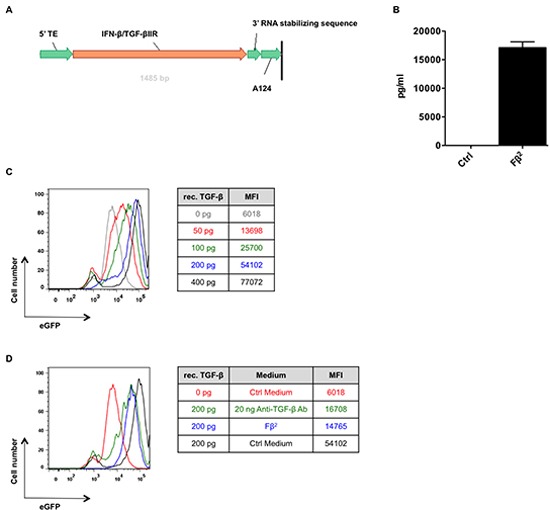
Fβ^2^ mRNA is translated to a functional protein **(A)** Schematic representation of the Fβ^2^ mRNA construct. **(B)** Splenocytes were cultured for 5 hours in Fβ^2^, control (Ctrl) supernatants or exposed to increasing amounts of recombinant IFN-β. qPCR was performed to determine *Mx1* expression. The graph depicts the amount of fusokine (pg/ml). The quantity was determined based on the expression of *Mx1* upon treatment with recombinant IFN-β and normalized to 2^−Δct^ for *Ppia* (n=3). **(C)** The TGF-β reporter HEK293T cell line was cultured for 24 hours in 0 to 400 pg/ml of recombinant TGF-β (rec. TGF-β). The histogram overlay depicts the eGFP expression. Representative plots are shown (n=4) **(D)** The TGF-β reporter HEK293T cell line was cultured for 24 hours in Fβ^2^, Ctrl supernatants or exposed to 20 ng of a commercial available anti-TGF-β antibody, and supplemented with 200 pg of recombinant TGF-β. The histogram overlay depicts the eGFP expression. Representative plots are shown (n=3).

To address whether mRNA encoding Fβ^2^, a fusion protein consisting of IFN-β and the ectodomain of the TGF-β receptor II (Fig. 1A), can be translated into a functional protein, we electroporated HEK293T cells with 20 μg of Fβ^2^ or eGFP mRNA. Supernatants were collected 24 hours later. First, we determined the functionality and estimated the amount of the *in vitro* generated fusokine. Therefore, we quantified the expression of the type I IFN-inducible gene *Mx1* in splenocytes cultured for 6 hours with the supernatants or with varying amounts of recombinant IFN-β. qPCR analysis demonstrated that the CT-values obtained upon treatment of splenocytes with Fβ^2^ supernatants was comparable to those obtained upon treatment of splenocytes with 17.1 ± 1.7 ng/ml (n = 3) recombinant IFN-β (Fig. 1B). To investigate the capacity of the fusokine to neutralize TGF-β we used the TGF-β reporter HEK293T cell line, which expresses eGFP under the control of a TGF-β responding promoter [11]. Indeed, higher eGFP expression was observed when the cell line was cultured with increased amounts of recombinant TGF-β (Fig. 1C). This was greatly reduced by the presence of Fβ^2^ in the supernatants (Fig. 1D). Additionally, the neutralization capacity of the fusokine was compared to a commercially available neutralizing anti-TGF-β antibody. The capacity of Fβ^2^ to neutralize TGF-β was comparable with 20 ng/ml of the commercially available anti-TGF-β antibody (Fig. 1D). These results are consistent with the quantity of Fβ^2^ estimated based on the expression of the IFN-inducible *Mx1* gene (Fig. 1B). Together, these data demonstrate that the mRNA encoding the Fβ^2^ fusokine is translated into a functional protein.

### The Fβ^2^ fusokine modulates myeloid cells to improve CD8^+^ T cell responses

**Figure 2:**
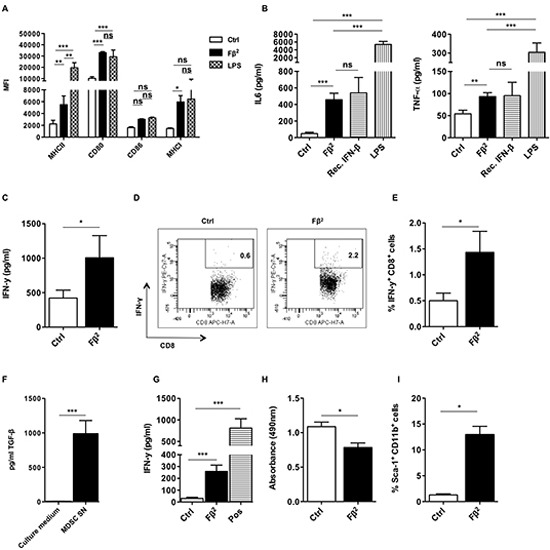
The Fβ^2^ fusokine modulates myeloid cells to improve CD8^+^ T cell responses (A-E) DCs were cultured for 48 hours in Fβ^2^ or Control (Ctrl) supernatants. **(A)** Expression of MHC II, CD80, CD86 and MHC I was evaluated by flow cytometry. The column graph depicts the median fluorescence intensity (MFI) (n=6). **(B)** Supernatants were analyzed for the presence of IL-6 and TNF-α (n=3-12). (C-E) DCs were pulsed with the peptide SIINFEKL and co-cultured for 6 days with CD8^+^ OT-I cells at a 1:10 ratio. **(C)** The production of IFN-γ by OT-I cells was determined by ELISA (n=9). **(D)** The production of IFN-γ by OT-I cells was confirmed via flow cytometry. Representative plots are show. **(E)** The column graph depicts the results of the experiments (n=6). **(F)** The TGF-β reporter HEK293T cell line was cultured for 24 hours in the conditioned medium used to generate MDSCs or in supernatants of *in vitro* generated day 5 MDSCs (n=3). (G-I) MDSCs were cultured for 3 days in Fβ^2^ or Ctrl supernatants. **(G)** These MDSCs were co-cultured for 3 days with CD8^+^ splenocytes that were activated with anti-CD3/anti-CD28 beads. Activated CD8^+^ splenocytes cultured in the absence of MDSCs served as a positive control (Pos). Supernatants were screened for the presence of IFN-γ (n=4). **(H)** MDSC viability was assessed using a colorimetric assay. The colorimetric assay reflects the amount of viable cells in the plate, and is measured as absorbance. The graph depicts the absorbance (n=3). **(I)** Flow cytometry was performed to assess the percentage of CD11b and sca-1^+^ cells (n=3).

To analyze the effect of Fβ^2^ on DCs, we cultured them for 48 hours in supernatants of HEK293T cells that were electroporated with 20 μg of Fβ^2^ or eGFP mRNA. DCs cultured with 20 ng/ml of recombinant IFN-β or activated for 4 hours with 100 ng/ml LPS were used as a control. Flow cytometry analysis revealed that DCs cultured with Fβ^2^ displayed an enhanced expression of co-stimulatory and antigen-presenting molecules (Fig. 2A), and secreted pro-inflammatory cytokines (Fig. 2B). To further evaluate the functionality of these DCs, we performed an *in vitro* stimulation of OT-I cells. We demonstrated that DCs pulsed with SIINFEKL peptide and cultured in the presence of Fβ^2^ lead to enhanced production of IFN-γ by antigen-specific CD8^+^ OT-I cells (Fig. 2C-D). We next analyzed the effect of Fβ^2^ on MDSCs. To that end, MDSCs that closely resemble those found within tumors were generated *in vitro* [12, 13]. Of note, these MDSCs produce high levels of TGF-β (Fig. 2E). The MDSCs were cultured for 3 days in supernatants of HEK293T cells that were electroporated with 20 μg of Fβ^2^ or eGFP mRNA. We found that MDSCs cultured in the presence of Fβ^2^ were no longer able to fully suppress the functionality of CD8^+^ T cells as shown by the ability of these T cells to produce IFN-γ (Fig. 2F). This might be explained by the reduced cell viability and the increased expression of the surface marker sca-1 on the MDSCs cultured in Fβ^2^ supernatants (Fig. 2G-I). Overall, these data suggest that Fβ^2^ potentiates the antigen-presenting function of DCs, whilst decreasing the suppressive capacity of MDSCs, therefore supporting CD8^+^ T cell-mediated responses.

### Tumor cells treated with Fβ^2^ show lower proliferation rates and increased expression levels of MHC I and PD-L1

**Figure 3:**
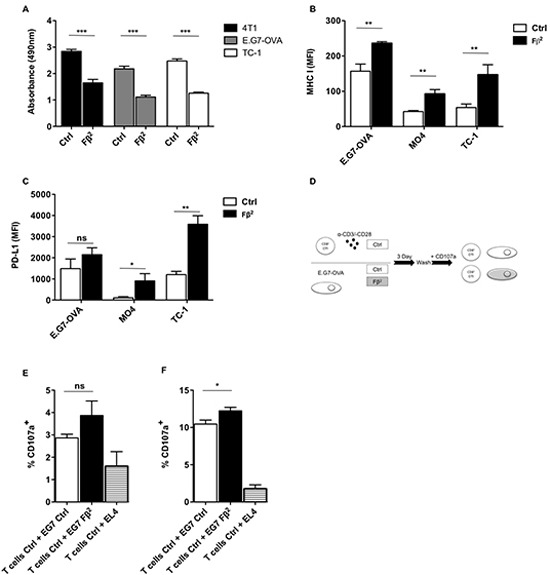
Tumors treated with Fβ^2^ show lower proliferative rates and express increased levels of MHC I and PD-L1 **(A)** 4T1, E.G7-OVA and TC-1 tumor cells were cultured for 4 days in Fβ^2^ or Ctrl supernatants. Cell proliferation was assessed using a colorimetric assay (n=3). (B-C) E.G7-OVA, MO4 and TC-1 cells were cultured for 24 hours in Fβ^2^ or Ctrl supernatants, after which expression of **(B)** MHC I and **(C)** PD-L1 was evaluated by flow cytometry. The column graphs depict the MFI (n=3). (D-F) E.G7-OVA tumor cells were cultured for 3 days in Fβ^2^ or Ctrl supernatants, after which they were co-cultured with CD8^+^ OT-I spleen cells in the presence of anti-CD107 antibodies for 4 hours at a tumor:T cell ratio of 1:10 **(E)** and 1:1 **(F)**. Four hours later cells were additionally stained for CD3 and CD8. The graphs depict the amount of CD107a and CD8^+^ T cells (n=3).

To investigate the effect of Fβ^2^ on tumor cells, we cultured tumor cells of various histological origin for 1 or 4 days with Fβ^2^ supernatants. Subsequently, we evaluated their phenotype and proliferation respectively. Tumor cells exposed to Fβ^2^ showed decreased proliferation (Fig. 3A) and enhanced expression of the antigen-presenting molecule MHC I as well as the co-inhibitory molecule PD-L1 (Fig. 3B-C). Next we analyzed whether exposure of E.G7-OVA cells to Fβ^2^ facilitated their recognition by activated CD8^+^ OT-I cells (Fig. 3D) despite PD-L1 up-regulation. These T cells showed an increased cytolytic activity (Fig. 3E-F), indicating that the expression of PD-L1 on tumor cells did not completely abrogate their recognition by T cells.

### Simultaneous exposure of tumor cells and CD8^+^ T cells to Fβ^2^ significantly enhances the killing capacities of antigen-specific T cells

**Figure 4:**
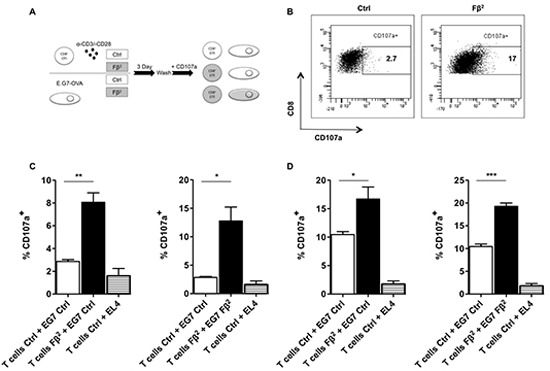
Simultaneous exposure of tumor cells and CD8 T cells to Fβ^2^ significantly enhances the killing capacities of antigen-specific T cells **(A)** CD8^+^ OT-I spleen cells were cultured for 3 days in Fβ^2^ or Ctrl supernatants. Simultaneously, E.G7-OVA tumor cells were cultured for 3 days in these supernatants. Subsequently, both cell populations were washed and mixed together as depicted. These were co-cultured in the presence of anti-CD107 antibodies for 4 hours at a tumor:T cell ratio of **(C)** 10:1 or **(D)** 1:1, after which the T cells were labeled with anti-CD3 and anti-CD8 antibodies. **(B)** The flow cytometry graphs are representative for the experiments. (C-D) The graphs depict the percentage of CD107a within CD8^+^ T cells in the co-cultures (n=3 and n=4, respectively).

To investigate the direct effect of Fβ^2^ on CD8^+^ T cells, we cultured CD8^+^ OT-I spleen cells for 3 days in supernatants of HEK293T cells that were electroporated with 20 μg of Fβ^2^ or eGFP mRNA. Simultaneously, E.G7-OVA tumor cells were cultured in Fβ^2^ containing supernatants. Subsequently, co-cultures were set up according to the scheme shown in figure 4A, and the degranulation ability of the CD8^+^ OT-I cells was evaluated (Fig. 4B-D). We demonstrated that CD8^+^ OT-I cells pre-treated with Fβ^2^ recognized tumor cells more efficiently and that this recognition was enhanced when tumor cells were pretreated with Fβ^2^ (Fig. 4C-D). In summary, we showed that the Fβ^2^ fusokine potentiates the killing capacity of CD8^+^ T cells.

### Intratumoral delivery of Fβ^2^ mRNA delays tumor growth

**Figure 5:**
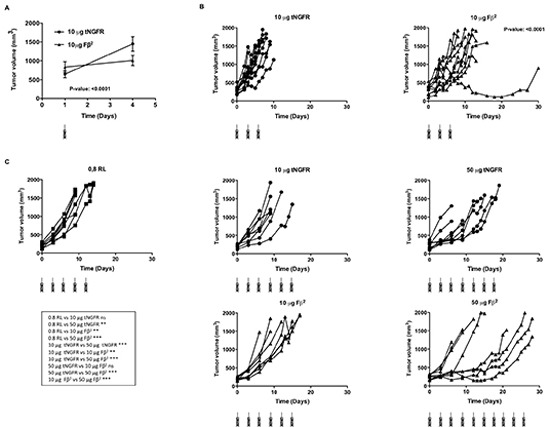
Intratumoral delivery of Fβ^2^ mRNA to E.G7-OVA bearing mice delays tumor growth (A-C) E.G7-OVA cells (5 × 10^6^) were transplanted subcutaneously in C57BL/6 mice. **(A)** Mice were treated with a single intratumoral injection of 10 μg mRNA encoding tNGFR (circle) or Fβ^2^ (triangle). The graph shows the average tumor size at the day of treatment and 3 days later for a total of 11 mice and summarizes the results of 4 independent experiments. **(B)** Mice (10 per group) were treated with 3 intratumoral injections of 10 μg of tNGFR (circle) or Fβ^2^ (triangle) mRNA at three-day intervals. The graph depicts the size of the tumor of individual mice. **(C)** Mice (8 per group) were treated with consecutive intratumoral injections of 0.8 RL (square), 10 or 50 μg of tNGFR (circle) or Fβ^2^ (triangle) mRNA at three-day interval. The graph depicts the size of the tumor of individual mice.

**Figure 6:**
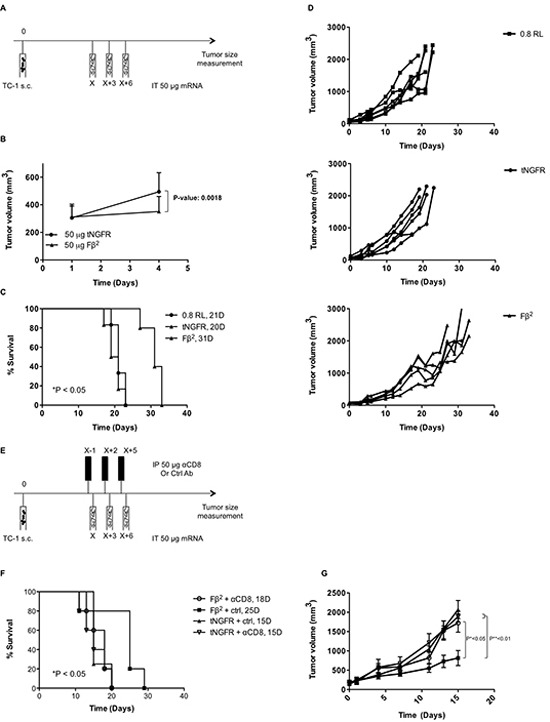
Intratumoral delivery of Fβ^2^ mRNA to TC-1 bearing mice delays tumor growth (A-G) TC-1 cells were transplanted subcutaneously in C57BL/6 mice. Once the tumors reached an average size of 100 mm^3^ the mice were treated according to the scheme shown in A. Tumor growth was monitored. **(B)** The graph shows the average tumor size at the day of treatment and 3 days later (n=11). (C-D) Mice (5-6 per group) were treated with 3 intratumoral injections of 0.8 RL (square), 50 μg of tNGFR (circle) or Fβ^2^ (triangle) mRNA at three-day intervals. **(C)** The graph depicts the median survival. **(D)** The graph depicts the growth of tumors in individual mice. **(E)** Treatment regimen with CD8^+^ T cell depletion. **(F)** The graph depicts the survival of the mice treated as in E. **(G)** The graph depicts the mean of the tumor sizes of the respective groups (5 mice per group).

To analyze the therapeutic potential of mRNA encoding the Fβ^2^ fusokine, we first treated mice bearing E.G7-OVA tumors with a single intratumoral injection of 10 μg of Fβ^2^ mRNA. Mice treated with a single injection of 10 μg tNGFR mRNA served as a control. Tumor growth was delayed over a period of three days (Fig. 5A), after which the effect was no longer detected (data not shown). Therefore, we subsequently treated tumor-bearing mice with three intratumoral injections of 10 μg Fβ^2^ or tNGFR mRNA at a three-day interval, which resulted in a prolonged survival of the mice (Fig. 5B). To further improve this therapeutic outcome, we further increased the dose of mRNA to 50 μg and delivered the mRNA continuously at a three-day interval (Fig. 5C). This regimen was compared to the delivery of 10 μg of mRNA. Mice treated with repeated injections of the vehicle (0.8 Lactated Ringer's solution) were used as a control. Notably, the treatment with 50 μg of tNGFR mRNA prolonged mice survival when compared to the treatment with 10 μg of tNGFR mRNA. In addition, we observed that in the groups treated with 10 μg of mRNA, the non-stop treatment regimen did not improve the survival as compared to the three-day regimen (Fig. 5B-C). Importantly, four out of eight mice treated with 50 μg of Fβ^2^ mRNA demonstrated a long-term survival when compared to mice treated with 10 μg Fβ^2^ mRNA or 50 μg of tNGFR mRNA (Fig. 5C). Based on these data, an optimal treatment regimen consisting of three immunizations at a three-day interval with 50 μg of mRNA was defined and then applied to the treatment of mice bearing TC-1 tumors (Fig. 6A). Consistent with the E.G7-OVA model, we observed an immediate and a prolonged delay in TC-1 tumor growth (Fig. 6B-C). Consequently, these mice showed an increased survival (Fig. 6D). To investigate the role of CD8^+^ T cells in this therapeutic outcome, we depleted this cell population prior and during the treatment (Fig. 6E). The results showed that CD8^+^ T cells are the predominant cell type involved in the observed antitumor activity, since their depletion abrogated the therapeutic effect of the Fβ^2^ fusokine (Fig. 6F-G).

### Blockade of the PD-1/PD-L1 interaction combined with Fβ^2^ mRNA treatment improves therapeutic responses

**Figure 7:**
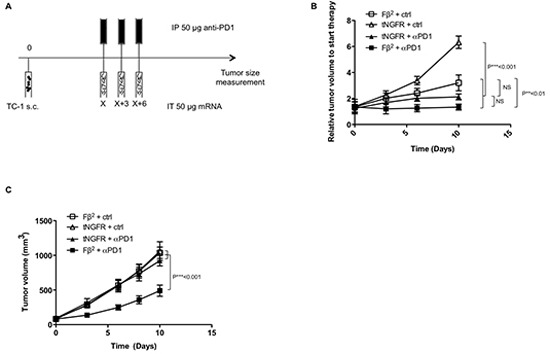
Blockade of PD-1/PD-L1 interactions combined with Fβ^2^ mRNA treatment improves therapeutic responses TC-1 cells were transplanted subcutaneously in C57BL/6 mice (5 per group). (A-C) Once the tumors reached a size of 100-500 mm^3^ mice were treated according to the scheme shown in A. Subsequently, tumor growth was monitored. **(B)** The graph depicts the mean of the tumor sizes of the respective groups when applying a less aggressive model using 2 × 10^4^ TC-1 cells. **(C)** The graph depicts the mean of the tumor sizes of the respective groups when applying a more aggressive model using 2 × 10^5^ TC-1 cells.

Although intratumoral injection of Fβ^2^ mRNA induces therapeutic responses, we demonstrated both *in vitro* (Fig. 3C) and *in vivo* (data not shown) that Fβ^2^ mRNA induces PD-L1 up-regulation. We therefore wondered whether additional targeting of the PD-1/PD-L1 pathway could improve therapeutic responses. To test this, we combined intratumoral delivery of Fβ^2^ mRNA with three intraperitoneal injections of anti-PD1 monoclonal antibodies (Fig. 7A-B). This resulted in an inhibition of TC-1 tumor growth (Fig. 7B). We next increased the amount of tumor cells injected, hereby mimicking a faster growing tumor setting. In this case, no effect was observed for the single treatment with Fβ^2^ mRNA or anti-PD1 alone (Fig. 7C). However, the combination of Fβ^2^ mRNA and anti-PD1 resulted in a delayed tumor growth and thus in an increased survival.

## DISCUSSION

In this study, we demonstrated the feasibility of intratumoral delivery of mRNA encoding immunomodulating proteins to fine-tune the tumor environment and confer antitumor immunity. More specifically, we evaluated the ability of a fusokine called Fβ^2^, composed of IFN-β and the ectodomain of the TGF-β receptor II.

Over the years, much effort has been put into priming and generating highly potent tumor-specific CTLs by means of cancer vaccination [14]. Although this strategy leads to the priming of tumor-specific CTLs [15], these T cells display reduced functionality due to exposure to the inhibitory mechanisms at the tumor site, which might explain poor results in the control of established tumors upon cancer vaccination. Several approaches are currently under development to tackle this problem. The success of anti-CTLA-4, anti-PD-L1 and anti-PD-1 antibodies in the treatment of various cancer types undoubtedly supports the idea of targeting inhibitory mechanisms that drive CTL dysfunctionality [16–18].

Here we evaluated the use of mRNA encoding the fusokine Fβ^2^ as a tool to deliver proteins with immunomodulating capacity. This work builds on the previous finding that mRNA can be delivered to the tumor and is engulfed by DCs [4, 5]. Based on these results we hypothesized that DCs could be exploited as ‘factories’ for the production of immunomodulating proteins. Moreover we prove the feasibility of using mRNA encoding a novel fusokine Fβ^2^, consisting of IFN-β and the ectodomain of the TGF-β receptor II. The combination of IFN-β and a TGF-β signaling antagonist was recently shown to have promising antitumor effects [19].

Type I IFNs have been described as stimulants of DC differentiation and maturation [20, 21]. In addition, it was recently demonstrated by Fuertes *et al* [22] that IFN-β plays an essential role in priming of T cells by attracting CD8α^+^ DCs to the tumor environment. This subset of DCs is important for the cross-presentation of tumor antigens to CD8^+^ T cells and lack of this population results in defective CD8^+^ T cell immune responses. Importantly, Van Lint *et al* proved that CD8α^+^ DCs are the main cell population involved in the uptake of mRNA in the tumor [5]. We demonstrated that Fβ^2^ induces DC activation. Surprisingly, Fβ^2^ matured DCs did not secrete enhanced IL-12 levels (data not shown). It has been shown that IL-6 secretion by DCs stimulated with type I IFNs plays a major role in the protection of T cell effector functions from the suppressive capacity of Treg cells [23]. In accordance with their maturation status, these DCs were able to stimulate antigen-specific CD8^+^ T cells.

Tumors have the capacity to take advantage of myeloid cell plasticity in order to skew them towards an immunosuppressive phenotype [24], which is confirmed by the presence of MDSCs within tumors. To study the effects of Fβ^2^ on MDSCs, we generated these cells *in vitro* as it was recently shown that large numbers of MDSCs that closely resemble MDSCs found within the tumor, could be induced [12, 13]. This resemblance was phenotypically based on *inter alia* the high expression of PD-L1, Arg-1 and CD86 and the lower expression of CD62L [13]. This is of a particular importance given difficulties in obtaining high numbers of pure MDSCs from tumors. Moreover it was also established that splenic MDSCs from tumor-bearing mice are phenotypically and functionally distinct from tumor MDSCs [24]. In accordance with previous reports, we demonstrated that *in vitro* generated MDSCs secrete high levels of TGF-β, a characteristic feature of *in vivo* tumor-infiltrating MDSCs [12, 13]. This local accumulation of TGF-β was shown to be accompanied with different immunosuppressive cell types, making TGF-β a core soluble factor in skewing the immunosuppressive balance [25–27]. In addition, type I IFNs have direct effects on the phenotype and function of myeloid cells. TGF-β antagonists and type I IFNs hamper MDSC-mediated suppressive functions [28, 29]. This effect could be linked to the induction of apoptosis as shown by Ellermeier *et al* [29]. MDSCs exposed to both TGF-β blockade and type I IFNs display an increased expression of CD11c, CD80 and a decrease in the granulocytic fraction [28, 29]. Interestingly, sca-1 up-regulation on MDSCs inversely correlates with their suppressive capacity [29], and our fusokine increases the expression of this marker on treated MDSCs.

Consistent with a previous study showing that MHC I on tumor cells is up-regulated upon treatment with IFN-β, we demonstrated that Fβ^2^ enhanced MHC I expression [30]. Loss of MHC I expression on cancer cells allows them to escape immunosurveillance, by making them invisible to CD8^+^ T cells [31]. By enhancing MHC I expression, Fβ^2^ showed to increase tumor cell recognition by CD8^+^ T cells. This was confirmed in a co-culture of CD8^+^ OT-I cells with tumor cells, exposed or not to the Fβ^2^ fusokine. However, the increase in tumor cell recognition was moderate, which might be explained by the enhanced PD-L1 expression on tumor cells treated with Fβ^2^ [32, 33]. Up-regulation of PD-L1 is a major obstacle in IFN-β based cancer immunotherapy as it can lead to T cell anergy and apoptosis through interaction with PD-1 [34, 35], but can also down-modulate T cell effector activities [36]. Targeting of this PD-1/PD-L1 interaction in type I IFN based therapy was shown to improve the therapeutic outcome [37, 38]. Importantly, we demonstrated that recognition of tumor cells pretreated with Fβ^2^ was augmented when T cells were exposed to Fβ^2^. Taken together, our *in vitro* findings point out that delivery of Fβ^2^ to the tumor environment might lead to a ‘CTL-supporting’ rather than a ‘CTL-suppressive’ environment. This was further confirmed by our *in vivo* data. We showed that intratumoral delivery of Fβ^2^ mRNA results in a delayed tumor outgrowth. The benefit of the Fβ^2^ mRNA therapy was dependent on CD8^+^ T cells, as the antitumor effect of the therapy was abrogated when mice were depleted of this cell population. We showed that the prolonged delay in growth was dependent on the repeated delivery of Fβ^2^ mRNA. The latter is consistent with the findings of Narumi *et al* [23], who indicated that low levels of type I IFNs were needed for at least 10 days to obtain successful antitumor immunity. Finally, we demonstrated *in vivo* that additional targeting of the PD-1/PD-L1 interaction improved the Fβ^2^ therapy, confirming the detrimental impact of PD-1 engagement on T cells in the tumor environment.

In conclusion, we established the feasibility of using mRNA encoding immunomodulating proteins to modulate the tumor environment. We provide evidence that Fβ^2^ works via a multistep process acting on DCs, MDSCs, CD8^+^ T cells as well as tumor cells. Its effect can be further enhanced through additional blockade of PD-1/PD-L1 interactions. This study supports therefore the paradigm that combining immunotherapeutic antitumor strategies might tackle the immunosuppressive tumor environment and lead to an improved outcome [39].

## MATERIALS AND METHODS

### Mice

6–12 week old female C57BL/6 mice (Charles River, Wilmington, USA) were housed and handled according to the regulations of the Animal Care Committee of the Vrije Universiteit Brussel (VUB). OT-I mice that carry a transgenic CD8^+^ T cell receptor (TCR) specific for the MHC class I-restricted ovalbumin (OVA_257–264_) peptide SIINFEKL were provided by B. Lambrecht (University of Ghent, Ghent, Belgium).

### Cell lines and Reagents

The human embryonal kidney (HEK) 293T, the breast cancer 4T1, the melanoma B16-F0 and the T-cell lymphoma E.G7-OVA cell lines were obtained from the American Type Culture Collection (Rockville, Maryland, USA). The TGF-β reporter HEK293T cell line was previously described [40]. The mouse melanoma cell line MO4 and the lung carcinoma cell line TC-1 were kindly provided by K. Rock (University of Massachusetts Medical Center, Massachussetts, USA) and T.C. Wu (Johns Hopkins Medical Institution, Baltimore, Maryland, USA). The recombinant IFN-β was purchased from Biolegend. The neutralizing anti-TGF-β antibody was purchased from eBioscience (clone 1D11.16.8). The anti-CD8 (2.43), anti-PD1 (J43) and isotype control (Hamster IgG) monoclonal antibodies were purchased form BioXCell (New Hampshire, USA).

### mRNA production

mRNA encoding Fβ^2^ (1485 base pairs) is comprised of a 5′ terminal cap, the genetic sequence encoding a fusion protein consisting of mouse IFN-β and the ectodomain of TGF-β receptor II, a 3′ RNA stabilizing sequence and a poly(A) tail. The pEtheRNA-Fβ^2^ vector used to produce the mRNA encoding the Fβ^2^ fusokine (produced by eTheRNA, Kortenberg, Belgium) was linearized at the 3′ end of the poly(A) tail using the restriction enzyme *Bsp* M1. The mRNA was produced by *in vitro* transcription as previously described [41]. The production of mRNA encoding the reporter eGFP or truncated nerve growth factor receptor (tNGFR) was previously described [41].

### Electroporation of HEK293T cells with mRNA

Electroporation of HEK293T cells with mRNA was performed according to the protocol described for mRNA electroporation of DCs [42].

### Intratumoral administration of mRNA

C57BL/6 mice were inoculated subcutaneously with 5 × 10^5^ E.G7-OVA cells, 2 × 10^4^ or 2 × 10^5^ TC-1 cells. Tumors with a volume of 100–500 mm^3^ were injected intratumorally with 50 μl of the indicated amount of mRNA dissolved in 0.8 Lactated Ringer's solution (0.8RL).

### RNA isolation, cDNA synthesis and real-time PCR

Total RNA was isolated using the SV Total RNA Isolation System (Promega, Madison, USA). The extracted RNA was reverse transcribed into cDNA with the RevertAid^TM^ H Minus First Strand cDNA Synthesis Kit (Fermentas Inc., Maryland, USA) using the random hexamer primers. Samples were subjected to real-time PCR analysis on an ABI PRISM 7700 Sequence Detection System (Applied Biosystems, Life Technologies, Ghent, Belgium). Primers for amplification of *Mx1* were as follows: 5′-CGAGAAGTCCGGAAGCTTGT-3′ and 5′-CATGTACTGAGAAGTTTCATGCA-3′; Probe FAM-CAATTGCCGTCACCGTTCGTTTTCA -TAMRA (Eurogentec, Seraing, Belgium). Primers for the amplification of peptidylprolyl isomerase A (*Ppia*) were as follows: 5′-TTCACCTTCCCAAAGACCAC-3′ and 5′CAAACACAAACGGTTCCCAG-3′; Probe FAM-TGCTTGCCA/ZEN/TCCAGCCATTCAG-3IABkFQ (Integrated DNA Technologies, Leuven, Belgium).

### *In vitro* generation of dendritic cells and myeloid-derived suppressor cells

Bone marrow-derived DCs were generated and activated with 20 ng/ml recombinant IFN-β or 100 ng/ml lipopolysaccharide (LPS) as described [42].

Bone marrow-derived MDSCs that resemble tumor MDSCs were generated as described [12]. Briefly, bone marrow cells were collected from femur and tibia, treated with red blood cell lyses buffer and washed with Dulbecco's Phosphate Buffered Saline (DPBS, Sigma-Aldrich, Zwijndrecht, Belgium). Bone marrow cells were cultured in 75% conditioned medium obtained from mouse GM-CSF secreting B16-F0 cells and 25% Iscove's Modified Dulbecco's Medium (IMDM, Lonza, Verviers, Belgium). The culture was supplemented with 10% fetal clone I (Harlan, Horst, the Netherlands), 100 U/ml penicillin, 100 μg/ml streptomycin and 2 mM L-Glutamine (Sigma-Aldrich). The MDSC cultures were refreshed at day 3 and 5 and ready for use at day 5–7.

### *In vitro* OT-I stimulation assay

*In vitro* generated DCs were pulsed with 5 μg/ml SIINFEKL (Eurogentec) for 90 minutes at 37°C in a humidified incubator. Subsequently, DCs were co-cultured at a 1:10 ratio with CD8^+^ T lymphocytes that were enriched from the spleen of OT-I mice using the CD8a^+^ T cell Isolation Kit II (Miltenyi Biotec, Gladbach, Germany). Supernatants of HEK293T cells that were electroporated with mRNA encoding eGFP or Fβ^2^ were added to the co-cultures. On day 6, supernatants were collected and analyzed via ELISA for IFN-γ production (eBioScience, San Diego, California, USA). GolgiPlug-containing medium was added and intracytoplasmatic staining for IFN-γ was performed 24 hours later.

### CD8^+^ T lymphocyte suppression assay

CD8^+^ T lymphocytes were isolated from the spleen of C57BL/6 mice using the CD8α^+^ T cell Isolation Kit II and were co-cultured with *in vitro* generated MDSCs at the indicated ratios in the presence of anti-CD3/anti-CD28 antibody coated microbeads (Invitrogen, Oslo, Norway) and 100 U/ml IL-2 (Peprotech, Rocky Hill, New Jersey, USA). Non-stimulated CD8^+^ T lymphocytes and CD8^+^ T lymphocytes stimulated in the absence of MDSCs were used as a control. Three days after the start of the co-culture the supernatants were collected and the production of IFN-γ was analyzed by ELISA (eBioScience).

### CD107a assay

Isolated CD8^+^ OT-I cells were stimulated with anti-CD3/anti-CD28 coated microbeads and cultured in Fβ^2^ or control supernatants for three days. Simultaneously E.G7-OVA tumor cells were cultured in Fβ^2^ or control supernatants and EL4 cells were used as a negative control. After three days, the cells were thoroughly washed, mixed and co-cultured in the presence of fluorescein isothiocyanate (FITC)-conjugated CD107a antibodies (Becton Dickinson [BD], Erembodegem, Belgium). Four hours later, the cells were washed and additionally stained for CD3 and CD8, after which all samples were acquired on the LSRFortessa (BD).

### Flow cytometry

Staining of cell surface markers was previously described [43]. The following antibodies were used: FITC-conjugated antibodies against CD11b (BD), AlexaFluor647 (AF647)-conjugated antibodies against CD11c and Ly6G (BioLegend, San Diego, California, USA), PE-conjugated antibodies against CD4 (BD), CD11b, MHC II (BioLegend), CD25, PD-L1 and PD-1 (eBioScience), PE-Cy7-conjugated antibodies against Ly6C (BioLegend), PercP-Cy5.5-conjugated antibodies against CD3 (BioLegend) and CD4 (BD), Pacific Blue-conjugated antibodies against CD3 (BioLegend), Brilliant Violet 605-conjugated antibodies against CD45 (BD), APC-conjugated antibodies against Foxp3 (eBioScience), APC-H7-conjugated antibodies against CD8 (BD), Brilliant Violet-conjugated antibodies against CD3 (BioLegend) and FITC-conjugated MHC class I (H2-K^b^, prepared in-house), PE-Cy7-conjugated antibodies against IFN-γ (eBioScience). Data were collected using the FACSCanto or LSRFortessa Flow Cytometer (BD) and analysis was performed with FACSDiva (BD) or Flow Jo 7 software (Tree Star Inc., Ashland, Oregon, USA).

### Enzyme-linked Immunosorbent Assay

Supernatants were screened in a sandwich ELISA for the presence of IL-6, IL-12, TNF-α or IFN-γ (eBioscience).

### Colorimetric assay (MTS assay)

To assess the viability of MDSCs and tumor cell proliferation, an MTS (3-(4,5-dimethylthiazol-2-yl)-5-(3-carboxymethoxyphenyl)-2-(4-sulfophenyl)-2H-tetrazolium) assay was performed according to the manufacturer's protocol (Promega, Wisconsin, USA).

### Statistical analysis

Comparison of two data sets was performed using the unpaired student's t-test. For the comparison of more than three groups, we performed a one-way ANOVA followed by a Bonferroni's multiple comparison test. Number of asterisks in the figures indicates the level of statistical significance as follows: *, *p < 0.05*; **, *p < 0.01*; ***, *p < 0.001*. The results are shown in a column graph or table as the mean ± standard error of the mean (SEM). Survival was visualized in a Kaplan-Meier plot. Differences in survival were analyzed by the log-rank test.
